# The ABCA1 blocking agent probucol decreases capacitation in ejaculated dog spermatozoa

**DOI:** 10.1186/s13028-019-0500-2

**Published:** 2020-01-06

**Authors:** Sabine Schäfer-Somi, Sven Budik

**Affiliations:** Platform for AI and ET, Department for Small Animals and Horses, Vetmeduni Vienna, Veterinärplatz 1, 1210 Vienna, Austria

**Keywords:** ABCA1, Cholesterol transport, Dogs, Probucol, Spermatozoa

## Abstract

**Background:**

The ATP binding cassette (ABC) transporters participate in the cholesterol and phospholipid transport within and through cell membranes of many cells including spermatozoa. Cholesterol efflux is important for capacitation of spermatozoa. ABCA1 expression has been assessed in canine spermatozoa previously but its role in capacitation still has to be determined. The aim of the study was to test whether inhibition of ABCA1 (1) decreases capacitation in ejaculated and epididymal canine sperm samples and (2) decreases cholesterol efflux in the same samples. Twenty-one ejaculates and sperm from 22 epididymal tails were collected from healthy dogs. Motility was measured by CASA and viability assessed after staining with SYBR-14/PI. Samples from ejaculated sperm and sperm from epididymal tails were aliquoted. One part was incubated with the ABCA1 inhibitor probucol, the other served as a negative control. In all samples, capacitation was evaluated by chlortetracyclin (CTC) assay and cholesterol was measured by cholesterol efflux assay and colorimetric enzymatic assay.

**Results:**

In ejaculated sperm, blockade of ABCA1 with 100 µM of probucol/mL of sample resulted in a significantly higher percentage of uncapacitated and acrosome reacted spermatozoa (P < 0.001 and P = 0.031), capacitation was significantly decreased (35% in probucol samples vs 54.2% in controls, P < 0.001). In probucol inhibited sperm samples from epididymal tails, the percentage of capacitated spermatozoa did not differ between groups but the percentage of acrosome reacted spermatozoa increased significantly (P = 0.014). The cholesterol measurement revealed significantly lower cholesterol concentration in the probucol group when compared to the controls (P = 0.035), however only in ejaculated sperm samples.

**Conclusions:**

CTC assay and cholesterol measurement revealed significant differences between groups; we conclude that inhibition of ABCA1 significantly decreased capacitation and cholesterol efflux in ejaculated canine spermatozoa. The inhibition was not complete but ABCA1 is supposed to contribute to capacitation in canine ejaculated spermatozoa. ABCA1 is probably not important for capacitation of epididymal spermatozoa but might exert other functions during spermatozoa ripening.

## Background

The ATP binding cassette (ABC) transporters are known to participate in the cholesterol and phospholipid transport within and through cell membranes of many different cells, including spermatozoa [1–3]. However, no study have investigated the function in canine spermatozoa. ABC transporters are present in spermatozoa membranes of many species. ABCG2 is expressed in mature spermatozoa from mice, rats, bulls and humans [4]. In cattle, ABCG2 was found to be functional and to participate in cholesterol efflux; however in epididymal sperm samples only [5]. In mice, expression of ABCA1, A7, A17 and G1 was detected [1, 6].

ABCA1 is highly expressed in murine testicular tissue as well [7] and is supposed to regulate lipid transport. Recently expression of ABCA1 was assessed in human spermatozoa membranes and an important role in spermatozoa development was suggested [8]. Cholesterol efflux is an essential step during capacitation of spermatozoa. Leahy and Gadella [9] presented a model for the reverse cholesterol transport (RCT) from the spermatozoa membrane to high-density lipoproteins (HDL). The ABC transporters are believed to transport cholesterol to certain micro-domains (rafts) in the outer spermatozoa membrane, mainly located in the apical region of the head, where the cholesterol molecules are accumulating and imported into HDL molecules. These molecules can transport cholesterol to sites of need [9]. However, this is a model and might not be applicable for all ABC transporters. Single members of this transporter family have to be examined or to be functionally grouped; which ABC transporters participate in this cholesterol redistribution seems to be species-specific and to our knowledge so far no data were available for the situation in dog spermatozoa.

The ABCA1 transporter mainly transports cholesterol [10]. Previously we assessed abundance of ABCA1 in canine spermatozoa, mainly in the acrosome and midpiece [11], while ABCG1 could not be detected in canine spermatozoa membranes. In a subsequent study we determined in bad freezers a lower percentage of spermatozoa with ABCA1 signal in the acrosome and a higher percentage of spermatozoa with complete loss of ABCA1 than in good freezers [12]. We hypothesized that this might be caused by capacitation induced by cold and osmotic stress.

The lipid-lowering drug probucol inhibited cholesterol efflux in human skin fibroblasts and J774 mouse macrophages [13–15], however, no study exists investigating the effect on spermatozoa membranes. In the present study we therefore tried to block the ABCA1 transporter protein in canine spermatozoa membranes by using different concentrations of this specific ABCA1 inhibitor [13].

In dogs, the ABCA1 transporter was assessed in both epididymal and ejaculated spermatozoa, even though at different sites [11]. Since epididymal spermatozoa undergo functional changes after ejaculation, different effects of ABCA1 inhibition would be possible before and after ejaculation.

The aim of this study was thus to investigate, whether ABCA1 participates in cholesterol transport and capacitation of canine ejaculated and spermatozoa from epididymal tails by blocking ABCA1 with its specific inhibitor probucol.

## Methods

### Study design

**Fig. 1:**
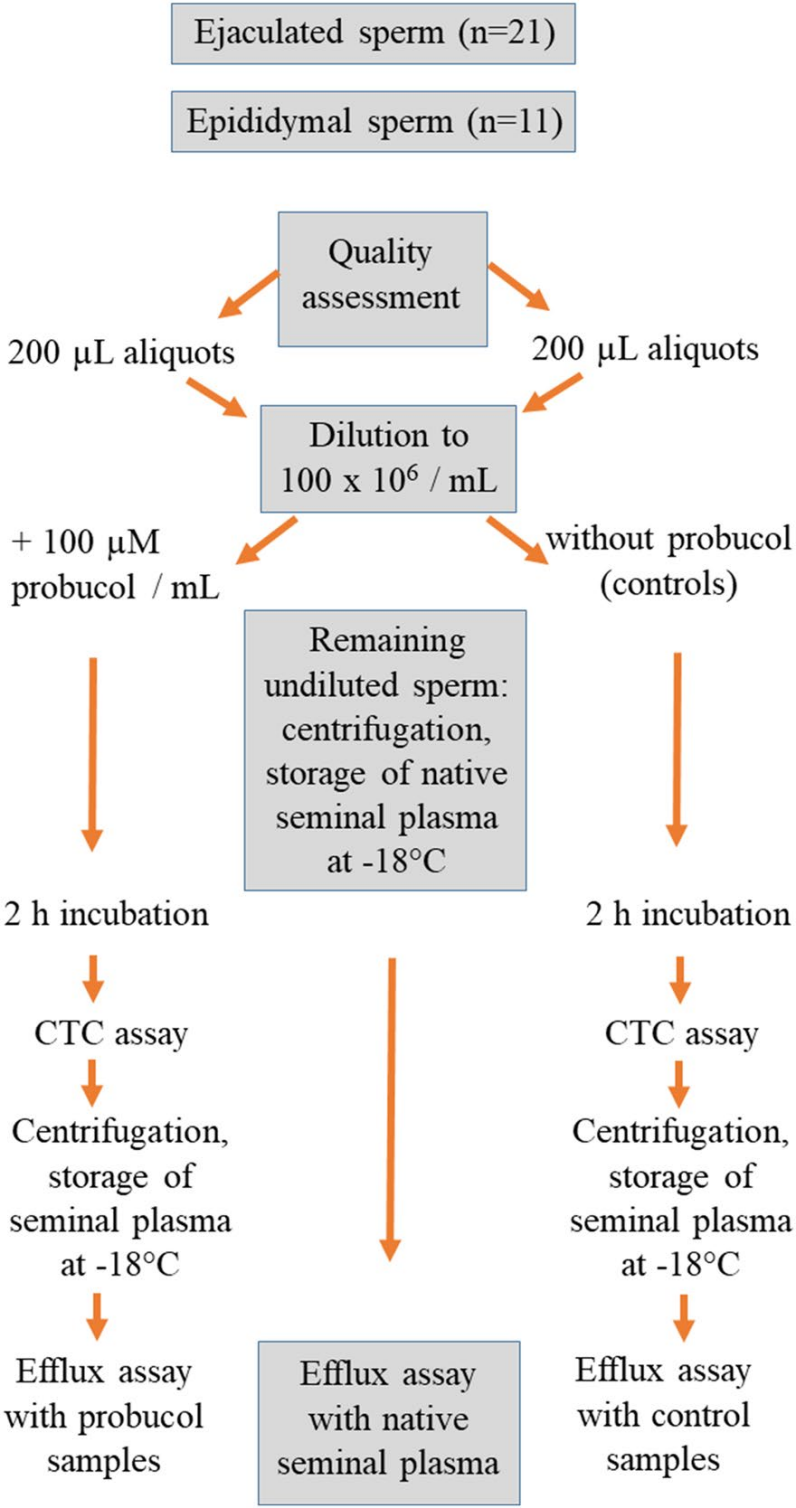
Experimental scheme. All semen samples (ejaculated sperm and epididymal sperm) underwent a quality assessment. Ejaculated and sperm from epididymal tails were equally treated and examined. Each sperm sample was aliquoted (n = 2 aliquots per group, 200 µL each), diluted and incubated with probucol (experimental sample) or without probucol (control sample). Remaining raw sperm samples were centrifuged and the seminal plasma/cauda epididymal fluids stored for cholesterol measurement. After incubation, experimental and control samples were centrifuged and the pellet further processed for CTC assay. The supernatant was stored for cholesterol measurement

The study design is illustrated in Fig. 1.

### Semen collection and quality assessment

Twenty-one ejaculates were manually collected from 6 healthy stud dogs with proven fertility and of different breeds: Malinois (n = 1; 5 ejaculates), Beagle (n = 2; 13 ejaculates), Flat Coated Retriever (n = 2; 2 ejaculates), Brazilian Terrier (n = 1; 1 ejaculate); the average age was 4.5 ± 3 years. The dogs either belonged to the Clinic for Obstetrics, Gynecology and Andrology, Vetmeduni Vienna or were introduced as ambulant patients for semen conservation. Only the sperm rich fraction of samples with good semen quality [16] was used for the experiments. All samples first underwent quality assessment as described in detail by [12].

### Motility assessment

Motility (M), progressive motility (P) and further motility parameters were examined by CASA (CASA, Sperm Vision^®^, Minitüb, Tiefenbach, Germany) after dilution to 100 × 10^6^ spermatozoa /mL with a TRIS-citric acid fructose buffer [17–19]. The temperature of analysis was 37 °C. A 3 µL droplet was placed on a glass slide and covered with a cover slip. Seven fields were evaluated per sample with 100 cells per field and a frame rate of 60/sec, always by the same examiner. The following motility parameters were assessed: Curvilinear velocity (VCL, µm/sec), Linear velocity (VSL, µm/sec), Mean velocity (VAP, µm/sec), Mean coefficient (STR, %), Linear coefficient (LIN, %), Wobble coefficient (WOB, %), Frequency of head displacement = beat cross frequency (BCF, Hz), Amplitude of lateral head displacement (ALH, µm), Distance curved line (DCL, µm), Distance straight line (DSL, µm), Distance average path (DAP, µm). In the CASA, total motility was defined as VCL > 15 µm/sec, progressive forward motility as VCL > 15 µm/sec and LIN > 0.9%. Linear motility was defined as STR > 0.9% and LIN > 0.5%.

### Viability assessment

The viability was assessed by using SYBR-14/PI double staining [19] visualized by an epifluorescence microscope connected to the CASA; briefly, 100 µL of sperm sample were placed in a vial with 2 µL of SYBR-14/PI and incubated for 10 min at room temperature (around 21 °C) in darkness. One droplet was placed on a glass slide, covered with a glass coverslip and evaluated via fluorescence microscopy at magnification × 400 (Olympus AX70, Olympus Optical Co., Ltd., Japan; U-MWB filter block, BP420-480 excitation filter, BA515 suppressor filter, dichromatic mirror: DM500). The heads of viable spermatozoa show bright green colour, damaged membranes are stained red. Both colours are recognized automatically by a video camera. Each sample was evaluated once, but at least 500 spermatozoa were evaluated per sample and the mean calculated by the software provided by SpermVision^®^. Results are given as mean percentage of spermatozoa with intact (green) membranes.

### Concentration measurement

The concentration of the sperm samples was determined using a Nucleocounter SP-100 (ChemoMetec A/S, Allerod, Denmark) according to the manufacturer’s instructions. The percentage of spermatozoa with abnormal morphology and acrosome integrity were assessed after fixation in Hancock’s solution (37% formalin; [20]) by analysing 200 cells per sample under a phase contrast microscope at × 1000 magnification (oil immersion). Acrosome and head abnormalities as well as pathological alterations of the midpiece and tail were recorded. In case of multiple sperm defects, the one with larger impact on fertilization was noted. Abnormalities of the acrosome and head were given priority.

### Incubation of semen samples with probucol

Inhibition of ABCA1 was performed with probucol (sigma Aldrich, P9672, Vienna, Austria), a specific ABCA1 inhibitor [13]. The probucol concentration was chosen according to the results of a pilot study investigating the effect of 10, 50 and 100 µM of probucol/mL of dog sperm sample on capacitation by using a chlortetracycline (CTC) assay. Only with 100 µM probucol/mL, a significant effect on capacitation but not on the viability was observed after 2 h of incubation; the latter was examined by using CYBR-14/PI after the incubation with and without probucol (data not shown). Therefore for the experimental series only the 100 µM solution was used. First a 10 mM stock solution was prepared: 5.16 mg probucol were dissolved in 1 mL 100% DMSO and aliquots were stored at − 18 °C.

Each semen sample was diluted with PBS to a density of 100 × 10^6^ cells/mL. Two aliquots of 200 µL each were taken, since this was the volume needed for the CTC assay that was performed immediately thereafter. One aliquot was supplemented with probucol to a final concentration of 100 µM/mL sperm sample (0.01 mg probucol/200 µL of sperm; 2 µL of the stock solution per 200 µL of sperm), the other served as control. The remaining raw sperm was centrifuged at 900*g* 5 min and the seminal plasma stored at − 18 °C for later cholesterol measurement. After incubation of experimental and control samples for 2 h [13], a CTC assay was performed.

### CTC assay and seminal plasma sampling

A CTC assay was performed as described by Hewitt [21] and Witte et al. [22] with experimental and control samples. All chemicals were obtained from Sigma-Aldrich (Vienna, A; Hoechst 33,258: H6024; glutaraldehyde: G6403; DABCO: D2522, chlortetracycline CTC: 94,498; Cysteine: 30,095; polyvinylpyrrolidone PVP: 81,400). CTC-solution: 4 mg CTC Chloride + 8.8 mg Cysteine in 10 mL CTC buffer: 24 mg TRIS + 76 mg NaCl/10 mL aqua bidest. PVP 2% in PBS: 40 mg PVP/2 mL PBS. The CTC buffer and PVP solution were aliquoted and stored at − 18 °C, the CTC solution was freshly prepared before each assay.

**Fig. 2:**
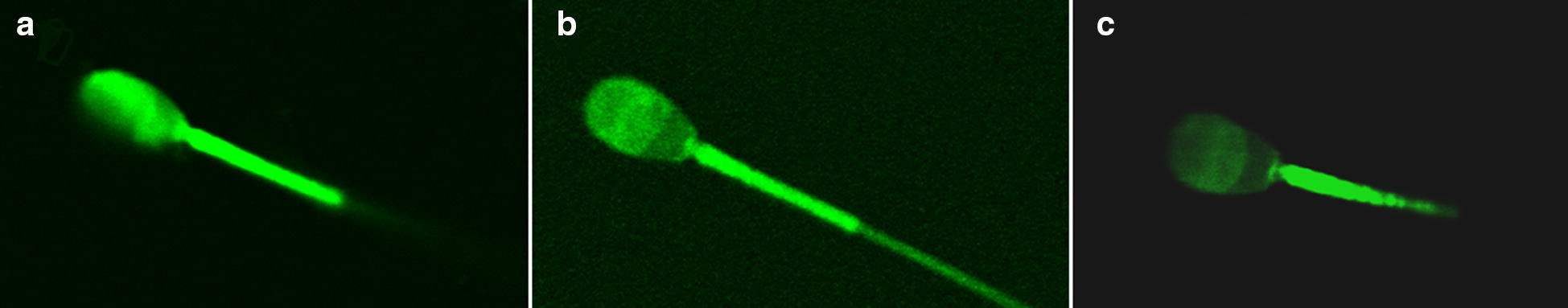
Uncapacitated, capacitated and acrosome reacted spermatozoa as visualized by CTC assay. For the CTC assay, all samples were evaluated under a fluorescence microscope, and the percentage of uncapacitated, capacitated and acrosome reacted spermatozoa assessed. **a** Uncapacitated spermatozoon (U), **b** capacitated spermatozoon (C), **c** acrosome reacted spermatozoon (AR)

After 2 h of incubation, 0.1386 µg Hoechst 33,258 (0.7 µg/mL) were added to both of the 200 µL aliquots and the samples incubated for 2 min. Afterwards, 2 mL PVP 2% in PBS were added and the mixture centrifuged at 900 g for 5 min. The supernatant was collected and stored at -18 °C for the cholesterol efflux assay performed later. The sperm pellet was mixed with 45 µL of CTC-solution and incubated for 20 s. After addition of 8 µL of a glutaraldehyde-solution 12.5%, 10 µL of each semen sample were put on a glass slide together with 1 droplet of DABCO 0.22 M in glycerol, mixed, the smear covered and sealed with clear varnish. The smears were stored at 4 °C in darkness and evaluated the next day. The percentage of uncapacitated (U), capacitated (C) and acrosome reacted (AR) sperm was assessed using a fluorescence microscope at magnification × 1000 (oil immersion, filter cube U-MWB; Fig. 2 a–c).

### Cholesterol measurement

The supernatant, obtained after washing 200 µL of sperm samples in PVP 2% in PBS, was used for measurement of cholesterol concentrations in the probucol treated and the control samples. Before measurement, the native seminal plasma without additives was diluted 1:50 with 1 × assay buffer (5 × assay buffer: 20 mL of 0.5 M potassium phosphate, pH 7.4, 0.25 M NaCl, 25 mM cholic acid, 0.5% Triton^®^ X-100; 2.5 mL of this solution were added to 10 mL deionized water to obtain the 1 × buffer), the probucol inhibited samples and the controls without probucol (supernatant) 1:10–1:20.

The *cholesterol efflux assay* (Amplex Red Cholesterol Assay Kit^®^ (Molecular probes, Eugene, OR, USA, cat no A12216) was performed according to the description of the manufacturer and the fluorescence measured using a TECAN Sunrise™ Genios fluorescence microplate reader (Tecan Austria GmBH, Grödig, Austria) and the Magellan™ software for calculation of data; excitation was at 520 nm and fluorescence detection at 590 nm. Background fluorescence determined for the no-cholesterol control reaction (1 × assay buffer), was subtracted from each value. Each sample was measured in double and the average calculated. The sensitivity of the assay was given as 0.2069 µM/L. The intra-assay coefficient of variation (CV) was 4.7%, the inter-assay CV was 5.7%. All results are given in µM/L and as average values ± SD.

The results were proven by measurement of cholesterol with a *colorimetric enzymatic assay* (Cobas 6000/c501, Cobas^®^, Roche diagnostics, Germany; sensitivity: 0.1–20.7 mM/L; 3.86–800 µg/mL): cholesterol esters are cleaved by the cholesterol esterase to yield free cholesterol and fatty acids; cholesterol oxidase then catalyses the oxidation of cholesterol to cholest-4-en-3-one and hydrogen peroxide. In the presence of peroxidase, the hydrogen peroxide affects the oxidative coupling of phenol and 4-aminophenazone to form a red quinone-imine dye; the colour intensity of the dye is directly proportional to the cholesterol concentration. All results are given in µM/L and as average values ± SD.

### Collection and treatment of epididymal sperm

Cauda epididymides were obtained after routine castration under general anaesthesia by cutting them off with a scalpel blade after removal of the testicular membranes. Each epididymal tail was placed in a petri dish, freed from blood, sliced with a scalpel blade and overlaid with 1 mL of PBS, thus 2 mL of the sperm suspension was collected per dog (n = 11 samples). After quality assessment as described before, the samples were treated and examined as the ejaculated sperm; a CTC assay was performed from each sample, and the cholesterol concentration in supernatant from probucol and control samples measured using the Amplex Red Cholesterol Assay Kit (Molecular Probes, Eugene, OR, USA, cat no A12216) and the colorimetric enzymatic assay as described for the ejaculated sperm.

### Statistics

IBM SPSS statistics version 24 (SPSS Ltd, Hong Kong) was used. Normal distribution of data was examined by using Kolmogorov Smirnov Test. Comparison between sperm samples inhibited with probucol and controls, and between ejaculated and epididymal sperm, respectively, was done by using a mixed model analysis. To exclude a possible dog effect while considering the repeated sampling in some cases, the dog was chosen as random factor, and all others as fixed variables. The mixed model analysis revealed that the dog effect was negligible.

Matched‐pair analyses, including the paired *t* test and the Wilcoxon test, were used to compare normally and non-normally distributed data between groups, where indicated. Pearson product-moment correlation coefficient (PPMCC) was used to test for linear correlations between selected data pools. All data are given as average values ± standard deviation (x ± SD). A p value of P < 0.05 was considered statistically significant.

## Results

Sperm samples from epididymal tails showed significantly less total motility (P < 0.001) and less viability (P = 0.001), but more abnormalities concerning the acrosome (P < 0.001), mid piece and total percentage of morphologically abnormal spermatozoa (P < 0.001) than ejaculated sperm (Table 1).

**Table 1:** Ejaculated and epididymal sperm—descriptive statistics

	Ejaculated (n = 21) X ± SD	Epididymal (n = 11) X ± SD	P =
Conc. raw × 10^6^	451.5 ± 101.0	158.8 ± 129.0	0.012
Conc. final × 10^6^	97.7 ± 6.1	88.9 ± 28.3	0.423
M %	92.7 ± 0.7	71.0 ± 11.8	0.000
P %	85.3 ± 4.1	58.8 ± 14.0	0.000
VAP µm/s	116.3 ± 10.1	77 ± 22.6	0.000
VCL µm/s	179.8 ± 19.3	153 ± 43.9	0.024
VSL µm/s	105.4 ± 9.3	61.8 ± 24.4	0.000
DAP µm	51 ± 4.17	35 ± 9.9	0.001
DCL µm	79.1 ± 8.3	70 ± 19.7	0.190
DSL µm	46.3 ± 3.9	28.1 ± 10.7	0.000
STR %	0.9 ± 0.01	0.7 ± 0.08	0.002
LIN %	0.5 ± 0.05	0.4 ± 0.1	0.000
Viab %	92.5 ± 4.9	67.1 ± 11.0	0.000
Acr %	9.1 ± 0.9	29.4 ± 10.3	0.000
Total %	31.9 ± 3.2	77.9 ± 10.8	0.000

*Acr* acrosome damages, *Conc*. *raw* concentration of raw sperm, *conc. final* concentration after dilution, *DAP* distance average path, *DCL* distance curved line, *DSL* distance straight line, *Ejaculated* ejaculated sperm, *Epididymal* sperm samples from epididymal tails, *LIN* linear coefficient, *M* motility, *P* = progressive motility, *STR* mean coefficient, *Total* percentage of morphologically abnormal sperm, *VAP* mean velocity, *VCL* curvilinear velocity, *Viab* viability, *VSP* linear velocity

### Ejaculated sperm

**Fig. 3:**
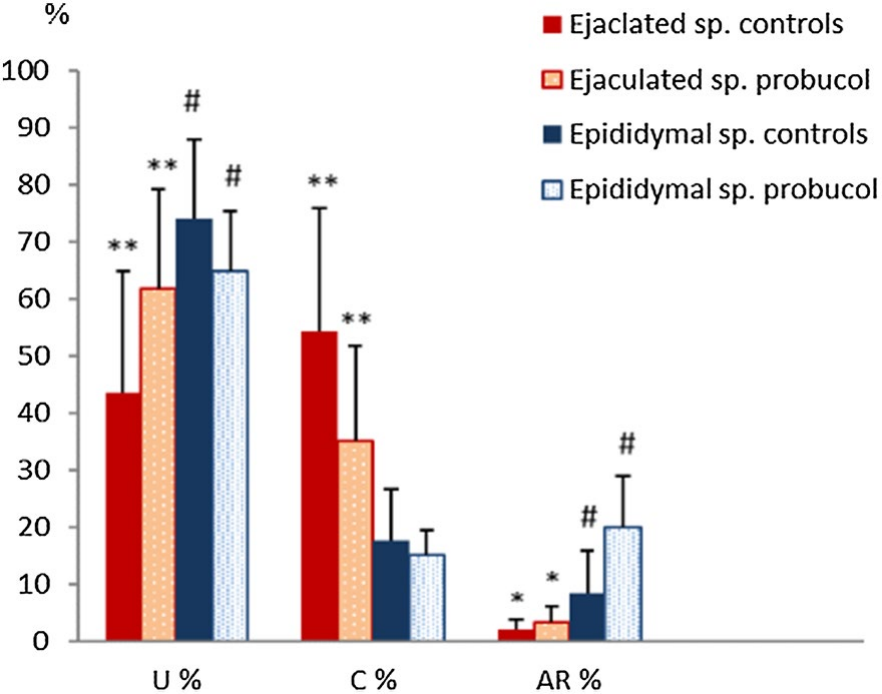
Results of the CTC assay: mean percentage of capacitated and acrosome reacted spermatozoa in controls, and samples inhibited with probucol. *U* uncapacitated sperm, *C* capacitated sperm, *AR* acrosome reacted sperm; *probucol* samples after addition of 100 µM of probucol/mL of semen, *controls* samples not treated with probucol, *Sp.* spermatozoa. Data are average values + SD. Bars with equal indices in one group differ significantly **P < 0.01, *^,#^ P < 0.05

Between the control group (Co) and the probucol treated group (Pro) significant differences were determined. After inhibition with probucol, a significant higher number of spermatozoa were uncapacitated and a lower number of sperm were capacitated (Fig. 2b) than in the controls (35% vs 54.2%, P < 0.001, Fig. 3). Furthermore, an increase in acrosome reactions by 1.3% was noted (P = 0.031).

**Fig. 4:**
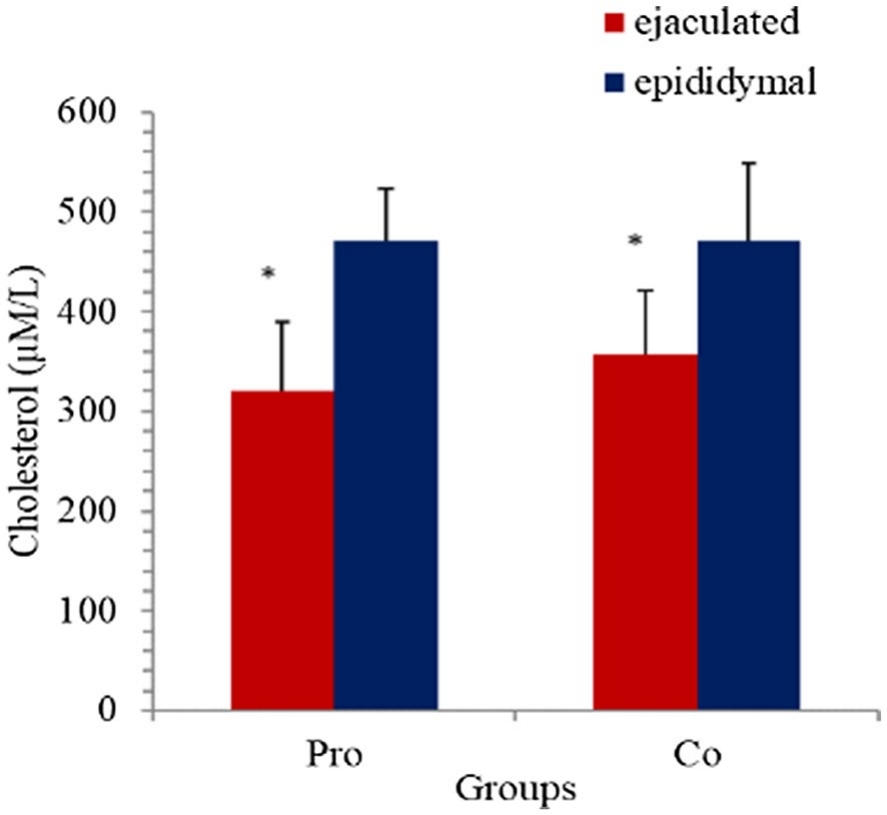
Results of the cholesterol efflux assay (colorimetric enzymatic assay). *Pro* samples after addition of 100 µM of probucol/mL of semen, *Co* samples not treated with probucol (controls), *ejaculated* ejaculated sperm, *epididymal* sperm from epididymal tails. Data are average values + SD. Bars with equal indices differ significantly, *P < 0.05

Measurement of the cholesterol concentration showed significantly lower cholesterol concentrations in the Pro group when compared to the controls (efflux assay: Pro: 20.86 ± 29.11 µM/L, Co: 23.41 ± 34.51 µM/L, P = 0.035; Results of the colorimetric enzymatic assay are shown in Fig. 4. Data of the two measurement methods correlated significantly (P < 0.001).

### Epididymal sperm

In the sperm samples from epididymal tails, inhibition with probucol increased the percentage of acrosome reacted spermatozoa (Fig. 2c) from 8.36% in the controls to 20% in the probucol treated samples (P = 0.014, Fig. 3), which significantly decreased the percentage of uncapacitated spermatozoa (Fig. 2a) in comparison to the non-treated control group (P = 0.047, Fig. 3). The percentage of capacitated spermatozoa did not differ significantly between groups.

The cholesterol concentration did not differ significantly between probucol and control group, with any assay used (efflux assay: Pro: 101.57 ± 64.93 µM/L, Co: 102.45 ± 55.25 µM/L; colorimetric enzymatic assay: Pro: 470.26 ± 104.64 µM/L, Co: 470.25 ± 155.98 µM/L, n.s., Fig. 4).

In seminal plasma from ejaculated sperm and in cauda epididymal fluids, cholesterol concentrations did not differ significantly; however, after addition of probucol a difference was seen in that ejaculated sperm revealed lower cholesterol concentrations than sperm from epididymal tails (efflux assay: ejaculated sperm: 20.86 ± 29.11, epididymal sperm: 101.57 ± 64.93 µM/L, P < 0.001).

## Discussion

Spermatozoa from epididymal tails already have the competence to move. In our study, immediately after collection and removal of the decapacitating factors, sperm samples from epididymal tails showed lower motility, viability and less intact acrosomes than ejaculated sperm samples; however, within the frame of normal when compared with the results from other studies [23, 24].

The inhibitor of the ABC transporter ABCA1, probucol, was used in previous studies for blockade of the cholesterol transport. Favari et al. [13] showed that in mouse macrophages, probucol impaired the translocation of ABCA1 from intracellular compartments to the plasma membrane; however, cholesterol efflux was inhibited up to 80% only, which might be due to the presence of additional ABC transporters [14, 15]. Probucol was found to be a highly effective ABCA1 blocking substance, but cell type and dose dependent. The effect on spermatozoa membranes has not been assessed so far. Therefore, during a pilot study we examined different concentrations of probucol in the sperm samples using the same stock solution as Favari et al. [13]; only with 100 µM/mL diluted sperm, an effect on capacitation but not on cell viability was seen in the CTC assay and after CYBR-14/PI stain, respectively.

We found that only in ejaculated sperm, probucol significantly affected capacitation and cholesterol efflux and not in samples from epididymal tails. The efflux was decreased but not completely inhibited; a reason for this phenomenon could be that in dog spermatozoa, additional cholesterol transporter systems with variable functionality exist; however, this remains to be proven.

At the first sight the result of the epididymal group seems to be contradictory but ABCA1 transporters are active transporters dependent among others on the energy status of the cell. Therefore blockade at different physiological statuses may lead to different results. In a former study [11], we detected expression of ABCA1 in canine testicular and epididymal tissue and spermatozoa membranes as has been done in human and stallion testicles, and spermatozoa [9, 25]. In canine spermatozoa, ABCA1 was mainly assessed in the tails of spermatozoa from the cauda epididymidis. There was a lack of ABCA1 abundance in the acrosome region, which might explain in part the lack of effect of the ABCA1 blockade on capacitation in spermatozoa from epididymal tails. Furthermore, the presence of other effective transport mechanisms might be causative or both. An explanation for the high percentage of AR in dog spermatozoa obtained from the cauda epididymidis could be that these cells obviously are able to deliver more cholesterol than dog spermatozoa after ejaculation. In our study, the cholesterol efflux was measured by using a proven cholesterol efflux assay, as has been done in other studies using different cells [13, 26] and in addition by means of colorimetric enzymatic assay. After inhibition with probucol, the cholesterol efflux was higher in sperm from epididymal tails than from ejaculated sperm; this could be explained by the maturation process in the different parts of the epididymis and different effects of the substance on the sperm membrane. Since high cholesterol concentrations might even inhibit capacitation [27], the increase in acrosome reactions might be spontaneous, as a natural event during storage of sperm samples at room temperature.

The activity of the ABCA1 transport system is influenced by the presence of cholesterol and external cholesterol acceptors like apolipoprotein or HDL; however, in this study, exactly the same medium was used for incubation of ejaculated sperm and sperm from epididymal tails. Different media therefore cannot explain the different effects of ABCA1 inhibition on cholesterol efflux and capacitation in ejaculated spermatozoa and spermatozoa from epididymal tails.

We measured significantly lower cholesterol concentrations in the group of ejaculated, probucol inhibited sperm samples than in the non-inhibited controls. Furthermore the CTC assay revealed clear differences, indicating changing Ca^2+^-distribution and a decreased transport of cholesterol in the diluted seminal plasma after ABCA1 blockade.

## Conclusions

Probucol caused a significant decrease in capacitation in ejaculated canine spermatozoa as confirmed by CTC assay and cholesterol measurement but is probably not important for capacitation of epididymal spermatozoa, or might play an underpart in this complex mechanism. However, consecutive investigations are necessary to recognize further possible cholesterol transport mechanisms for the efflux and uptake of cholesterol inside the canine sperm membrane, and the interaction with external cholesterol acceptors.

## Data Availability

The datasets used and/or analyzed during the current study are available from the corresponding author on reasonable request.
